# War and Bereavement: Consequences for Mental and Physical Distress

**DOI:** 10.1371/journal.pone.0022140

**Published:** 2011-07-12

**Authors:** Nexhmedin Morina, Ulrike von Lersner, Holly G. Prigerson

**Affiliations:** 1 Department of Clinical Psychology, University of Amsterdam, Amsterdam, The Netherlands; 2 Department of Psychology, Humboldt University, Berlin, Germany; 3 Dana-Farber Cancer Institute, Harvard Medical School, Boston, Massachusetts, United States of America; Federal University of Rio de Janeiro, Brazil

## Abstract

**Background:**

Little is known about the long-term impact of the killing of a parent in childhood or adolescence during war on distress and disability in young adulthood. This study assessed current prevalence rates of mental disorders and levels of dysfunction among young adults who had lost their father due to war-related violence in childhood or adolescence.

**Methods:**

179 bereaved young adults and 175 non-bereaved young adults were interviewed a decade after experiencing the war in Kosovo. Prevalence rates of Major Depressive Episode (MDE), anxiety, and substance use disorders, and current suicide risk were assessed using the Mini–International Neuropsychiatric Interview. The syndrome of Prolonged Grief Disorder (PGD) was assessed with the Prolonged Grief Disorder Interview (PG-13). Somatic symptoms were measured with the Patient Health Questionnaire. General health distress was assessed with the General Health Questionnaire.

**Findings:**

Bereaved participants were significantly more likely to suffer from either MDE or any anxiety disorder than non-bereaved participants (58.7% vs. 40%). Among bereaved participants, 39.7% met criteria for Post-Traumatic Stress Disorder, 34.6% for PGD, and 22.3% for MDE. Bereaved participants with PGD were more likely to suffer from MDE, any anxiety disorder, or current suicide risk than bereaved participants without PGD. Furthermore, these participants reported significantly greater physical distress than bereaved participants without PGD.

**Conclusion:**

War-related loss during middle childhood and adolescence presents significant risk for adverse mental health and dysfunction in young adulthood in addition to exposure to other war-related traumatic events. Furthermore, the syndrome of PGD can help to identify those with the greatest degree of distress and dysfunction.

## Introduction

Recent and ongoing wars and open conflicts have affected millions of civilians around the globe and about half of the survivors have been estimated to be under the age of 18 years [1], [2]. Research indicates enduring psychiatric sequelae in young and adult civilians of war and political violence even many years following war-related traumatic experiences [3]–[6]. Yet, a lack of information exists about the impact that the killing of a parent in childhood or adolescence in the context of war has on distress and disability in young adulthood.

In general, the death of a parent is regarded as one of the most stressful life events children and adolescents can experience [7], [8]. Loss of a parent in childhood has been shown to be associated with higher prevalence rates of mental disorders compared to rates observed in non-bereaved peers [9], [10]. There is also evidence that unnatural death of a parent during childhood and adolescence, such as parental suicide, possesses a stronger association with psychiatric sequelae than sudden natural parental death [10], [11]. These findings are consistent with research among adults indicating that bereaved individuals are more likely to suffer from physical and mental symptoms and to use health services more often than non-bereaved individuals [12].

In an effort to identify the most vulnerable bereaved individuals, clinical researchers have refined and tested diagnostic criteria for the syndrome of prolonged, or complicated, grief [13], [14]. The syndrome of prolonged grief has been shown to be distinct from Major Depressive Episode (MDE) and anxiety disorders [13], [14]. Symptoms of prolonged grief have also been distinctively related to response to treatment. Treatment with tricyclic antidepressants alone or in combination with interpersonal psychotherapy as used in the treatment of depression has proved to be ineffective [15], whereas psychotherapy designed specifically for treatment of symptoms of prolonged grief has shown effective results [16]. Furthermore, research has demonstrated incremental validity of prolonged grief [17]–[19]. Using samples of different type and temporal location in the course of grief as well as multiple measures of functioning and psychopathology, Bonanno et al. [17] recently offered additional, independent evidence demonstrating that grief symptoms are a unique predictor of psychopathology and functioning above and beyond symptoms of depression and post-traumatic stress disorder (PTSD) both cross-sectionally and longitudinally. Recent research has also provided psychometric validation for the criteria for Prolonged Grief Disorder (PGD) proposed to be included in DSM-5 and ICD-11 [20]. Research in North America and Western Europe has shown that symptoms of prolonged grief are associated with higher risk of suicidality and mortality, poorer physical health, greater use of health services, more psychological impairment and distress, as well as impairment in work and social functioning [12]. These data were evidence that lead to the proposed inclusion of PGD as an Axis I (Adjustment Disorder subtype) in DSM-5.

However, current findings on bereavement possess at least two limitations. First, most research on grief has been conducted among older bereaved individuals [20]. Second, there are relatively few published studies of bereavement using samples from countries outside North America and Western Europe. Against this background, we sought to examine the association between rates of mental disorders and dysfunction (i.e., suicide risk, somatic complaints, and general health distress) among young adults who, during middle childhood or adolescence, had lost their father due to war-related violence. Furthermore, we aimed at comparing these findings with a group of matched non-bereaved young adults. We decided to conduct the survey among a homogeneous group of bereaved young adult survivors of war and focused on war-related parental loss only because the impact of war-related killing of a parent in childhood or adolescence on disability and distress in adulthood has been neglected in previous research despite that fact that this type of bereavement is considered one of the most stressful life experiences during childhood or adolescence [7], [8]. Given that the majority of parents killed during a war were fathers, we focused on killing of the father. We did this not only for feasibility (i.e., more fathers available from which to sample) but because their deaths would probably be more directly connected to war (as opposed to being a civilian casualty), affect family financial security in a more uniform way, and would differ in important ways from that of the mother's death. Combining maternal and paternal deaths would, therefore, blur potentially important distinctions between them, so we opted to focus on paternal bereavement.

Based on bereavement research in general as well as on preliminary findings on the effect of war-related bereavement among adults [21], [22], we hypothesized that war-related bereavement constitutes a significant risk factor for distress and dysfunction above and beyond other war-related traumatic experiences. Additionally, we expected that the syndrome of PGD will be able to identify bereaved individuals at heightened likelihood for distress and dysfunction. We conducted our study with young survivors of the Kosovo war that started in mid-1998 and was ended by NATO air strikes a year later. This war has been described as leading to the worst ethnic cleansing in Europe since World War II [23].

## Methods

### Study Design

All interviews were conducted face-to-face between fall 2008 and summer 2009 (i.e. a decade after the war). The survey was conducted in cooperation with a psychosocial center for children and adolescents in the town of Gllogovc that enabled potential counseling for study participants in case they would feel distressed by the interview and seek professional counseling. Thus, the survey was conducted in the municipality of Gllogovc and three neighboring municipalities: Kline, Skenderaj, and Vushtrri. The three neighboring municipalities were randomly selected out of seven municipalities bordering Gllogovc. The total population of these four municipalities constitutes about 15% of the Kosovar population of about two million people. All participants were Kosovar Albanians as no other ethnicities live in the municipality of Gllogovc. The interviews were conducted face-to-face by five female psychologists who were experienced in conducting clinical interviews for a prior project [5]. In this prior project, inter-rater agreement among interviewers was assessed for the MINI in two mock interviews. Among 251 items, the mean agreement rate (i.e., all interviewers gave the same answer for each item) across two sessions was 90.2%.

### Ethics statement

Prior research with bereaved survivors of war in Kosovo had indicated that signed consent often leads to confusion among this population due to the fact that during the repression period people were forced by the police to sign declarations about false accusations [22]. Accordingly, in the current study, informed consent of participants was asked only in a verbal way. The interviewer documented whether potential participants stated verbally (1) that they had read the consent form and discussed it with the interviewer to their satisfaction and (2) that they agreed to participate in the study. Potential participants were informed that they could abandon the interview at any time if they felt disturbed by the any of the questions. Further, they were informed that if participation would cause distress, they could seek counseling at the cooperating psychosocial centre in Gllogovc. This concept procedure was approved by the ethics committee of the University of Amsterdam. All participants received five Euros as compensation for their participation.

### Participants

Potential participants needed to report exposure to at least one potential war-related traumatic event and no mental impairment due to an organic cause. In line with our objective to include participants who had lost the father during childhood or adolescence due to war-related violence, potential participants had to report a minimum age of six years and a maximum age of 18 years during the war. This criterion was chosen to make sure that participants had been old enough to remember the war (minimum age of six years) and yet still be living with their parents as the war had started (maximum age of 18 years). Potential participants were contacted directly at home without prior notification as telephone and postal communication services in Kosovo do not function effectively in the countryside. Furthermore, people in Kosovo have the habit of visiting each other without advance notice. Prior surveys also have contacted potential participants directly at home [24].

*Bereaved participants* had to report exposure to at least one potential war-related traumatic event and the loss of their father due to war-related violence. Lists of all families who had lost relatives during the war in the municipalities of Gllogovc, Kline, Skenderaj, and Vushtrri were provided by communal authorities. In each municipality, we approached 65 bereaved families. Out of 260 contacted participants, 67 did not meet the specified age criterion. Of 193 potential participants, 13 declined to participate in the study, indicating not wanting to talk about the war again or not having time for the interview. This resulted in a participation rate of 93.3% (180/193). Missing values resulted in the exclusion of one participant and leading to a total of 179 bereaved participants. Missing data were a minor problem in the study as a function of the interview format used to collect data.

*Non-bereaved participants* were contacted in exactly the same localities as bereaved participants. Potential participants had to report at least one potential war-related traumatic event and no loss of first-degree family members neither during nor after the war. We defined as first-degree relatives parents, siblings, and children. A random walk approach that has been reported in prior research with war survivors in the Balkans [5] was applied in this study. This involved random identification of streets in those localities where bereaved participants were contacted to recruit non-bereaved participants. On a particular street, every third house on the right was approached with a maximum of 15 interviews for that particular street. Out of 299 households contacted, 112 did not meet inclusion criteria (29 families reported loss of first-degree relatives after the war and 83 families did not meet the specified age criterion). Nine of 187 potential participants refused to participate in the study reporting not having time for the interview. The rate of participation among those who met inclusion criteria was 95.2% (178/187). Preliminary analysis revealed that three participants had reported death of first-degree family members after the war and were thus excluded from further analyses, resulting in a total sample size of 175.

### Measures

Socio-demographic characteristics of the samples were assessed using a brief structured questionnaire that has been used in prior research in Kosovo [5]. Potentially war-related traumatic events were measured using a checklist that assesses 18 potentially war-related traumatic events and has been used before in Kosovo [25].

MDE, anxiety disorders, suicide risk, and substance use disorders were assessed with the MINI International Neuropsychiatric Interview [26]. The MINI has shown similar diagnostic sensitivity compared to the Structured Clinical Interview for DSM-III-R and Composite International Diagnostic Interview [27], [28]. The MINI is the only structured psychiatric interview translated for use among Kosovar Albanians [29] and has been used several times among this population [5], [22], [30]–[32].

Prolonged Grief Disorder (PGD) was assessed with the structured Prolonged Grief Disorder Interview, the PG-13 [20], [22]. In this study, the criteria for the diagnosis of PGD were met if the respondent experienced for six months or longer post-death (a) separation distress at least once a day; (b) reported five or more out of nine cognitive, emotional, and behavioral symptoms at a “marked” or “overwhelming” level; and (c) the symptoms caused “marked and persistent dysfunction in social, occupational, or other important domains.”

Somatic symptoms for the last four weeks were measured with the Patient Health Questionnaire (PHQ-15) [33]. The 15 items of the PHQ-15 include 14 of the 15 most prevalent DSM-IV somatization disorder somatic symptoms. The PHQ-15 has shown good psychometric properties [33]. As prior research had indicated that the majority of Kosovar participants refuse to answer the two items of the PHQ-15 on “menstrual problems“ and “sexual problems” [34], these two items were omitted from the interview. In the current study, the internal consistency of the 13 items of the PHQ was α = .80 for the bereaved group and α = .72 for the non-bereaved group, respectively.

General health distress for the last four weeks was assessed with the 12-item version of the General Health Questionnaire (GHQ-12) [35]. This questionnaire has demonstrated good validity across different community and patient samples [36] and has also been used among the Kosovar population [34]. In the current study, the internal consistency of the GHQ-12 was α = .97 for the bereaved group and α = .73 for the non-bereaved group, respectively.

### Statistical Analysis

Analyses were conducted using SPSS V17. Prevalence rates of mental disorders were calculated as percentages of participants with a positive diagnosis at the time of the interview. Bivariate differences in socio-demographic characteristics, traumatic experiences, and prevalence of mental disorders between groups were evaluated using the *χ^2^* tests and t-tests were used depending on whether the variable was continuous, ordinal or categorical. Multivariate logistic regression analyses were used to examine associations between socio-demographic and war-related variables and mental disorders that significantly differed between bereaved and non-bereaved participants (MDE, PTSD, panic disorder, MDE or any anxiety disorder, and both MDE and PTSD). All variables were entered simultaneously in these analyses. Multicollinearity among potential predictor variables was assessed using the variance inflation factor (VIF) statistic from the equivalent linear regression model.

Incremental validity of the three most prevalent disorders (MDE, PTSD, and PGD) on dysfunction among bereaved participants was analyzed with hierarchical regression analysis. Here, number of war-related traumatic events and socio-demographic variables were entered at step one. PTSD and MDE status were entered at step two and PGD status was entered at step three.

## Results

### Socio-demographic and war-related characteristics

Socio-demographic and loss-related characteristics of the samples are reported in Table 1. Bereaved participants were more likely to have less formal education and were almost twice more likely to report unemployment than non-bereaved participants (both ps<.001).

**Table 1:** Socio-demographic variables of bereaved and non-bereaved participants.

	Bereaved participants(N = 179)	Non- bereaved participants(N = 175)	*t or X^2^*	*p-value*
Male gender	75 (41.9)	63 (36.0)	1.30	0.28
Age, M (SD)	20.3 (3.65)	20.0 (3.75)	1.16	0.25
**Education**			18.14	**<0.001**
Elementary	28 (15.6)	10 (5.7)		
High school	140 (78.2)	134 (76.6)		
University	11 (6.1)	31 (17.7)		
**Marital status**			4.26	0.24
Married	14 (7.9)	7 (4.0)		
Single	156 (87.6)	163 (93.7)		
Divorced	1 (0.6)	0 (0)		
Other	7 (3.9)	4 (2.3)		
**Employment status**			13.98	**0.001**
Training/education	89 (49.7)	114 (65.1)		
Employed	17 (9.5)	22 (12.6)		
Unemployed	73 (40.8)	39 (22.3)		
**Number of war-related loss of first-degree family in N (%)**			n.a.	n. a.
» 1	122 (68.2)	n.a.		
» 2	27 (15.1)	n.a.		
» 3 or more	30 (16.8)	n.a.		

*Note:* Data are given as the number (percentage) of participants unless otherwise indicated; n.a. = not applicable.

Among bereaved participants, nearly one third reported the killing of more than one first-degree relative during the war. Additionally, two participants reported death of first-degree relatives (brother) after the war.

Exposure to retrospectively reported war-related characteristics is shown in Table 2. Bereaved participants reported a significantly higher number of potentially war-related traumatic events (excluding loss of first-degree relatives). In particular, bereaved participants had experienced nine single war-related traumatic experiences significantly more often than non-bereaved participants (all ps<.05).

**Table 2:** Potentially traumatic war-related events of bereaved and non-bereaved participants.

	Bereaved participants(N = 179)N (%)	Non- bereaved participants(N = 175)N (%)	*X^2^*	*p-value*
Forced evacuation under violent threat	167 (93.3)	151 (86.3)	4.76	**0.029**
Lack of shelter	165 (92.2)	145 (82.9)	7.06	**0.008**
Lack of food or water	155 (86.6)	128 (73.1)	9.98	**0.002**
Combat situation	137 (76.5)	120 (68.6)	2.82	0.093
Forced separation from family members	137 (76.5)	94 (53.7)	20.31	**<0.001**
Repetitive house search by armed forces	134 (74.8)	105 (60.0)	8.91	**0.003**
Ill health without access to medical care	132 (73.7)	114 (65.1)	3.09	0.079
Being close to one's own death	119 (66.5)	78 (44.6)	17.21	**<0.001**
Torture	105 (58.7)	66 (37.7)	15.55	**<0.001**
Physical abuse	72 (40.2)	55 (31.4)	2.98	0.085
Serious injury	62 (34.6)	49 (28.0)	1.81	0.178
Kidnapped	61 (34.1)	27 (15.4)	16.47	**<0.001**
Imprisonment	40 (22.3)	16 (9.1)	11.58	**0.001**
Murder of stranger or strangers	26 (14.5)	16 (9.1)	2.45	0.127
Sexual abuse	6 (3.4)	3 (1.7)	0.96	0.328
Home displacement during war	120 (67.0)	110 (62.0)	3.28	0.194
Other war-related events	8 (4.5)	5 (2.9)	0.65	0.421
Number of war-related traumatic events excluding murder of family M (SD)	8.5 (2.3)	6.7 (3.3)	5.82	**<0.001**

*Note*: Data are given as the number (percentage) of participants unless otherwise indicated.

Pre- and post-war potentially traumatic events were reported only by a small proportion of participants and there were no significant differences between the samples.

### Prevalence of mental disorders and dysfunction among bereaved and non-bereaved participants

Observed prevalence rates of mental disorders and suicide risk of bereaved and non-bereaved participants are shown in Table 3a. Compared to non-bereaved participants, bereaved participants were significantly more likely to suffer from MDE, PTSD, and Panic Disorder. Accordingly, bereaved participants were significantly more likely to suffer from either MDE or any anxiety disorder as well as from comorbid MDE and PTSD than non-bereaved participants. There were no differences with regard to suicide risk between the two groups. Finally, not a single participant reported substance abuse or dependency.

**Table 3:** Current prevalence rates of mental disorders and suicide risk among the groups.

3a. Observed prevalence rates among bereaved and non-bereaved participants (N = 354)											
	MDE	PTSD	GAD	Social Phobia	Panic Disorder	Agoraphobia	OCD	PGD	MDE or any anxiety disorder	Both MDE and PTSD	Suicide risk
**Bereaved sample (n = 179)**	40 (22.3)	71 (40.0)	19 (10.6)	11 (6.1)	11 (6.1)	26 (14.5)	6 (3.4)	62 (34.6)	105 (58.7)	25 (14.0)	21 (11.7)
**Non-bereaved sample (n = 175)**	20 (11.4)	28 (16.0)	13 (7.4)	16 (9.1)	3 (1.7)	27 (15.4)	1 (0.6)	n.a.	70 (40.0)	5 (2.9)	11 (6.3)
**OR**(**95%CI**)	2.23(1.24–4.00)	3.45(2.09–5.71)	1.48(0.71–3.10)	0.65(0.29–1.45)	3.75(1.03–13.70)	0.93(0.52–1.67)	6.04(0.72-50-65)	n.a	2.13(1.39–3.25)	5.52(2.06–14.78)	1.98(0.93–4.24)
***P*** ** value**	**0.006**	**<0.001**	0.296	0.288	**0.032**	0.812	0.060	n.a.	**<0.001**	**<0.001**	0.074

3b. Observed prevalence rates among bereaved participants with or without PGD (N = 179)											
**PGD positive** **(N = 62)**	24 (38.7)	33 (53.2)	17 (27.4)	6 (9.7)	7 (11.3)	11 (17.7)	2 (3.2)	n.a.	47 (75.8)	16 (25.8)	16 (25.8)
**PGD negative** **(N = 117)**	16 (13.7)	38 (32.5)	2 (1.7)	5 (4.3)	4 (3.4)	15 (12.8)	4 (3.4)	n.a.	58 (49.6)	9 (7.7)	5 (4.3)
**OR**(**95%CI**)	3.98(1.91–8.30)	2.40(1.26–4.57)	21.90(4.84–98.99)	2.41(0.71–8.27)	3.63(1.01–12.95)	1.49(0.63–3.52)	0.95(0.17–5.36)	n.a.	3.19(1.61–6.33)	4.20(1.72–10.26)	7.78(2.70–22.49)
***P*** ** value**	**<0.001**	**<0.001**	**<0.001**	0.532	**0.001**	0.541	0.452	n.a.	**<0.001**	**<0.001**	**<0.001**

*Note*: Data are given as the number (percentage) of participants unless otherwise indicated; GAD = Generalized Anxiety Disorder; MDE = Major Depressive Episode; OCD = Obsessive Compulsive Disorder; PGD = Prolonged Grief Disorder; PTSD = Post-Traumatic Stress Disorder; n.a. = not applicable.

Multivariate logistic regression analyses examined associations between socio-demographic and war-related variables and mental disorders that significantly differed between bereaved and non-bereaved participants (see Table 4). The assessment of multicollinearity among potential predictor variables did not indicate serious multicollinearity (VIF exceeding 10). When all variables were entered simultaneously into the analyses, war-related loss of the father remained a significant predictor of MDE, PTSD, and Panic Disorder. Further, war-related loss of the father was also significantly associated with meeting criteria for either MDE or any anxiety disorder as well as for comorbid MDE and PTSD. Particularly, war-related loss of the father was the only significant predictor of MDE, Panic Disorder, and comorbid MDE and PTSD when all variables were considered. Additionally, PTSD was significantly associated with forced evacuation under violent threat. Finally, meeting criteria for either MDE or any anxiety disorder was significantly associated with less education, forced evacuation under violent threat, and war-related loss of the father.

**Table 4:** Predictors of MDE, PTSD, Panic Disorder, MDE or any anxiety disorder, and both MDE and PTSD among all participants (N = 354).

	MDE			PTSD			Panic Disorder			MDE or any anxiety disorder			Both MDE and PTSD		
	Adjusted OR(95% CI)		*P* value	Adjusted OR(95% CI)		*P* value	Adjusted OR(95% CI)		*P* value	Adjusted OR(95% CI)		*P* value	Adjusted OR(95% CI)		*P* value
**Education, y**	0.96	(0.85–1.09)	0.551	0.96	(0.87–1.07)	0.490	0.89	(0.70–1.13)	0.338	0.89	(0.81–0.99)	**0.030**	0.92	(0.79–1.08)	0.305
**Unemployed vs. employed or in training**	1.30	(0.69–2.45)	0.414	1.35	(0.78–2.33)	0.287	0.35	(0.09–1.46)	0.151	0.89	(0.54–1.48)	0.662	1.64	(0.71–3.82)	0.251
**Traumatic war events** **(Yes vs. No)**															
Forced evacuation under violent threat	1.89	(0.59–6.05)	0.284	4.06	(1.12–14.68)	**0.033**	1.89	(0.20–17.76)	0.579	3.85	(1.58–9.39)	**0.003**	1.12	(0.23–5.59)	0.886
Lack of shelter	0.58	(0.23–1.48)	0.257	3.09	(0.98–9.73)	0.054	0.49	(0.09–2.73)	0.418	1.49	(0.70–3.18)	0.307	2.66	(0.32–22.29)	0.367
Lack of food or water	2.24	(0.55–9.16)	0.263	0.56	(0.19–1.65)	0.291	0.69	(0.06–8.66)	0.772	0.54	(0.21–1.37)	0.192	2.46	(0.27–22.36)	0.423
Forced separation from family members	0.90	(0.29–2.87)	0.863	1.38	(0.48–4.00)	0.549	1.43	(0.09–22.24)	0.797	2.05	(0.85–4.94)	0.111	0.99	(0.16–5.93)	0.987
Repetitive house search by armed forces	0.70	(0.26–1.92)	0.493	1.40	(0.54–3.61)	0.490	0.91	(0.08–10.10)	0.941	1.12	(0.50–2.47)	0.788	1.50	(0.33–6.70)	0.599
Being close to one's own death	1.90	(0.79–4.56)	0.149	1.46	(0.73–2.93)	0.282	1.13	(0.20–6.47)	0.889	1.28	(0.71–2.30)	0.415	2.16	(0.58–8.09)	0.253
Torture	0.77	(0.32–1.81)	0.542	0.73	(0.35–1.54)	0.412	1.01	(0.16–6.55)	0.985	1.16	(0.60–2.24)	0.659	0.55	(0.18–1.68)	0.292
Kidnapped	1.25	(0.52–2.99)	0.622	1.14	(0.53–2.44)	0.738	0.61	(0.11–3.38)	0.567	0.86	(0.45–1.66)	0.654	2.15	(0.62–7.57)	0.231
Imprisonment	0.82	(0.34–1.97)	0.653	1.00	(0.46–2.17)	0.990	4.22	(0.51–34.79)	0.181	0.75	(0.38–1.51)	0.422	0.44	(0.14–1.32)	0.142
Father killed during war	2.07	(1.11–3.87)	**0.023**	2.94	(1.71–5.04)	**<0.001**	4.46	(1.14–17.44)	**0.032**	1.68	(1.06–2.67)	**0.027**	4.16	(1.49–11.58)	**0.006**

*Note:* MDE = Major Depressive Episode; PTSD = Post-Traumatic Stress Disorder.

Bereaved participants reported significantly higher scores of general health distress as measured with the GHQ (25.0 vs. 24.1, respectively; t = 2.11, p = .04). However, bereaved participants did not report higher scores of somatic complaints as measured with the PHQ than non-bereaved participants (18.4 vs. 17.8, respectively; t = 1.47, p = .14).

### Prevalence of mental disorders and dysfunction among bereaved participants with or without PGD

The prevalence rate of PGD among bereaved war survivors was 34.6%. Criteria for comorbid diagnoses of PTSD, MDE and PGD were met by 8.9% of all bereaved participants and 59.8% of them met criteria for any of these three disorders. Prevalence rates of mental disorders and suicide risk among bereaved participants with versus without PGD are reported in Table 3b. Bereaved participants with PGD were significantly more likely to suffer from MDE, PTSD, Generalized Anxiety Disorder, and Panic Disorder than bereaved participants without PGD. Furthermore, individuals with PGD were significantly more likely to report current suicide risk than those without PGD.

Bereaved survivors of war with PGD also reported significantly higher scores of somatic complaints (20.4 vs. 17.4, respectively; t = 4.44, p<.001) and general health distress (27.0 vs. 23.9, respectively; t = 4.49, p<.001) than those without PGD.

### Construct Specificity of PTSD, MDE, and PGD among bereaved participants

Three separate hierarchical regression models examined the predictive value of the three most prevalent disorders, PTSD, MDE, and PGD, on severity of suicide risk, general health distress, and somatic complaints. At step one, the number of war-related traumatic events, home displacement during war, gender, age, and years of schooling were entered. At step two, PTSD and MDE status were entered, whereas PGD status was entered at step three. Results are reported in Table 5. After controlling for model covariates, the diagnoses of MDE and PTSD were uniquely associated with greater severity of suicide risk, general health distress, and somatic complaints. When PGD was entered in the third step, it significantly predicted severity of suicide risk, general health distress, and somatic complaints over and above socio-demographic and war-related variables as well as PTSD and MDE (see Table 5). With the entry of PGD, only MDE and PGD (and not PTSD) were uniquely associated with greater general health distress and severity of suicide risk. With regard to somatic complaints, all three diagnoses independently predicted somatic complaints.

**Table 5:** Hierarchical regression models with PTSD, MDE, and PGD as predictors of general health distress, somatic complaints, and severity of suicide risk among bereaved participants (N = 179).

Outcome: severity of suicide risk							
Step		B	SE_b_	β	T	ΔR^2^	ΔF
1	number of traumatic events	.001	.01	.005	.07	.04	1.41
	home displacement during war	.12	.70	.13	1.75		
	gender	.13	.07	.13	1.75		
	age	−.01	.01	−.08	−1.00		
	years of schooling	.005	.01	.03	.73		
2	PTSD	.04	.07	.05	.60*	.17	13.79***
	MDE	.40	.08	.36	4.95***		
3	PGD	.23	.07	.23	3.12*	.22	9.70**

Outcome: general health distress							
Step		B	SE_b_	β	T	ΔR^2^	ΔF
1	number of traumatic events	.32	.14	.18	2.30*	.07	2.76*
	home displacement during war	.06	.68	.01	0.09		
	gender	1.73	.69	.19	2.50*		
	age	.11	.10	.09	1.14		
	years of schooling	−.11	.14	−.06	−.80		
2	PTSD	1.39	.63	.15	2.20*	.32	31.50***
	MDE	5.01	.73	.46	6.94***		
3	PGD	1.53	.65	.16	2.35*	.35	5.50**

Outcome: somatic complaints							
Step		B	SE_b_	β	T	ΔR^2^	ΔF
1	number of traumatic events	.29	.14	.16	2.12*	.08	2.95*
	home displacement during war	.18	.69	.02	0.26		
	gender	2.20	.70	.23	3.17**		
	age	.05	.10	.04	.46		
	years of schooling	−.07	.14	−.04	−.48		
2	PTSD	1.84	.63	.20	2.92*	.32	30.23***
	MDE	4.57	.72	.42	6.36***		
3	PGD	1.43	.64	.15	2.21*	.34	4.89*

*Note:* PTSD = Post-Traumatic Stress Disorder; MDE = Major Depressive Episode; PGD = Prolonged Grief Disorder.

*p<.05.

**p<.01;

***p<.001.

All *p*-values are two-tailed.

## Discussion

This study provides evidence that war-related loss of the father during childhood or adolescence uniquely contributes to distress and disability into adulthood over and above the experience of other war-related trauma. Results demonstrate that war survivors whose fathers had been killed during the war were significantly more likely to suffer from MDE, PTSD, and Panic Disorder as compared to non-bereaved war survivors. Accordingly, the majority of bereaved war survivors met criteria for either MDE or an anxiety disorder a full decade after the war. Additionally, one third of the bereaved participants met criteria for PGD while reporting that these symptoms caused persistent dysfunction in social, occupational, or other important areas of functioning. Among bereaved participants, the presence of PGD was associated with a higher prevalence rate of MDE or an anxiety disorder. Finally, survivors of war with PGD were significantly more likely to report current suicide risk than bereaved participants without PGD and to also report higher scores of somatic complaints and general health distress.

Given the lack of research on long-term effects of bereavement in childhood or adolescence as well as the lack of bereavement-related findings outside North America and Western Europe, the current study provides novel information on long-term sequelae of the violent death of the father during childhood or adolescence. The results are strengthened by the fact that both groups of participants were survivors of war and were recruited in exactly the same localities. Additional strengths of the study are that the interviewers were well trained with a relevant professional background and were familiar with the studied locations. Nevertheless, the study has a few limitations. Participants were recruited in only four municipalities and this might reduce the generalization of findings to other areas of war-related bereavement. Further, due to the cross-sectional format of the study, only point prevalence rates of the disorders were assessed and no definite conclusions on causal associations between the measured variables can be made. The reporting of traumatic experiences might have been influenced by recall bias with people with more current symptoms reporting more traumatic experiences [37]. Additionally, we did not assess whether fathers were killed as combatants or as civilians and neither did we assess whether participants were exposed to the killing of their father. Future studies need to investigate whether the role of the father during the war and experience of the killing of the father impact levels of dysfunction and distress following parental loss. Bereaved and non-bereaved participants were recruited in different ways (i.e., using a list provided by the communal authorities vs. a random sampling procedure), which might have lead to higher rates of mental disorders among bereaved participants than in the sample using random sampling. Furthermore, all interviewers were female and thus not matched to the gender of participants, which might have influenced reporting of sexual abuse. However, sexual abuse is more likely to have occurred among women than men and female participants were in fact interviewed by female interviewers. Moreover, prior studies with survivors of war in Kosovo have also reported extremely low rates of sexual abuse [5], [24], [34] indicating that report of sexual abuse might be strongly influenced by cultural stigma rather than gender of the interviewer. Future research needs to address these methodological approaches.

The results are consistent with other studies suggesting that war-related experiences can be associated with long-term psychiatric sequelae [3], [4], [6], [38]. A large-scale study with adults (mean age 39.4) conducted in Kosovo seven years after the war found a prevalence rate of MDE of 37.3% and a rate of PTSD of 18.2% [5]. The prevalence rate of MDE was higher in the Priebe et al. study than in both groups of the current survey. However, the study by Priebe et al. revealed that younger survivors of war reported lower rates of MDE than older participants and this might explain our finding of a lower prevalence rate of MDE in our younger sample. The higher prevalence rate of PTSD in our study, also as compared to Priebe et al. (40% vs. 18.2%), and the prevalence rate of PGD of 34.6% indicate that war-related traumatic events in conjunction with the killing of the father during childhood or adolescence are more likely to be associated with PTSD and PGD than MDE. These high prevalence rates are consistent with another study conducted seven years after the war among bereaved survivors of the Kosovo war (mean age 40.6) [22]. In this study by Morina et al., the prevalence rate of PTSD was 55% and that of PGD was 38.3%.

The fact that no participant reported any substance abuse disorder might appear surprising as research in other countries shows that people with a history of traumatic experiences report high rates of substance abuse disorders [39]. Yet, prior research in Kosovo has reported very low prevalence rates for substance abuse disorders. In the survey by Priebe et al. [5], the prevalence rate for substance dependence or abuse was 0.9% and the rate for alcohol dependency or abuse was 1.8%. Alcohol is little consumed in the areas where we recruited study participants that are inhabited exclusively by Muslim Kosovars. The fact that no positive cases with any substance abuse disorder were reported might further be explained by the strong stigmatization of people with substance abuse or dependency due to both religious and cultural factors in Kosovo. Fear of stigmatization might have led some study participants to conceal their substance abuse or dependency. It remains for future studies to clarify this issue.

Apart from higher prevalence rates of mental disorders, bereaved participants reported significantly lower education and a significantly higher unemployment rate. Lower education might be a consequence of the killing of the father. It might be a direct consequence of the absence of the father and the resultant need to take care of the family, which would leave less time and energy for pursuing education. It might also be a result of emotional difficulties related to the violent loss of the father in conjunction with other war-related experiences. Additionally, loosing the father often meant loosing the main, and mostly the only, breadwinner in the family. Finally, it is also likely to be a consequence of the absence of a father figure to support the child in pursuing education. Unemployment, on the other hand, might partly be due to a lower education. It is also likely that the higher unemployment rate among the bereaved group was associated with higher scores of adverse mental health [40].

The syndrome of PGD proved to be a good indicator of vulnerable bereaved individuals. As compared to bereaved individuals without PGD, those with PGD reported significantly higher prevalence rates of either MDE or any anxiety disorder, suicide risk, and higher scores of somatic complaints as well as general health distress. Additionally, PGD was uniquely associated with higher suicide risk, general health distress, and somatic complaints after controlling for war-related traumatic events, socio-demographic variable, and MDE and PTSD. These data are consistent with the literature on PGD [17], [20], [41] and thus support the proposal to include PGD in the next versions of the DSM and ICD.

In general, our findings demonstrate that the killing of the father during childhood and adolescence and the experience of other war-related events are associated with adverse health complaints and dysfunction during young adulthood. More importantly, while taking into account socio-demographic and war-related characteristics, the results yielded that war-related killing of the father can exacerbate the effects of other war-related traumatic experiences. The outcomes of the study highlight the need for long-term policies to meet the special mental health needs of bereaved war survivors.
